# Bisphenol S Disrupts Estradiol-Induced Nongenomic Signaling in a Rat Pituitary Cell Line: Effects on Cell Functions

**DOI:** 10.1289/ehp.1205826

**Published:** 2013-01-17

**Authors:** René Viñas, Cheryl S. Watson

**Affiliations:** Department of Biochemistry and Molecular Biology, University of Texas Medical Branch Galveston, Texas, USA

**Keywords:** bisphenol S, ERα, ERK activation, JNK activation, membrane estrogen receptors, nongenomic effects, prolactinoma cell line, xenoestrogens

## Abstract

Background: Bisphenol A (BPA) is a well-known endocrine disruptor that imperfectly mimics the effects of physiologic estrogens via membrane-bound estrogen receptors (mERα, mERβ, and GPER/GPR30), thereby initiating nongenomic signaling. Bisphenol S (BPS) is an alternative to BPA in plastic consumer products and thermal paper.

Objective: To characterize the nongenomic activities of BPS, we examined signaling pathways it evoked in GH_3_/B_6_/F_10_ rat pituitary cells alone and together with the physiologic estrogen estradiol (E_2_). Extracellular signal-regulated kinase (ERK)– and c-Jun-N-terminal kinase (JNK)–specific phosphorylations were examined for their correlation to three functional responses: proliferation, caspase activation, and prolactin (PRL) release.

Methods: We detected ERK and JNK phosphorylations by fixed-cell immunoassays, identified the predominant mER initiating the signaling with selective inhibitors, estimated cell numbers by crystal violet assays, measured caspase activity by cleavage of fluorescent caspase substrates, and measured PRL release by radioimmunoassay.

Results: BPS phosphoactivated ERK within 2.5 min in a nonmonotonic dose-dependent manner (10^–15^ to 10^–7^ M). When combined with 10^–9^ M E_2_, the physiologic estrogen’s ERK response was attenuated. BPS could not activate JNK, but it greatly enhanced E_2_-induced JNK activity. BPS induced cell proliferation at low concentrations (femtomolar to nanomolar), similar to E_2._ Combinations of both estrogens reduced cell numbers below those of the vehicle control and also activated caspases. Earlier activation of caspase 8 versus caspase 9 demonstrated that BPS initiates apoptosis via the extrinsic pathway, consistent with activation via a membrane receptor. BPS also inhibited rapid (≤ 1 min) E_2_-induced PRL release.

Conclusion: BPS, once considered a safe substitute for BPA, disrupts membrane-initiated E_2_-induced cell signaling, leading to altered cell proliferation, cell death, and PRL release.

Xenoestrogens (XEs) are a diverse group of synthetic agents (e.g., pesticides, surfactants, plastics monomers) that can mimic and disrupt the actions of physiologic estrogens (Colborn et al. 1993; Le and Belcher 2010; McLachlan 2001; Soto et al. 1994). Many XEs can remain in the environment for a long time, thus increasing the likelihood for human and wildlife exposure (Ahel et al. 1993; deJager et al. 1999; Dekant and Volkel 2008; Judson et al. 2010).

Bisphenol A (BPA), a leachable monomer of polymerized polycarbonate plastics, has been used commercially since 1957 (Bisphenol A Global Industry Group 2002) and is also found in food can liners and coatings on thermal cash register paper (Zalko et al. 2011). Humans are exposed to BPA primarily from food and water (H_2_O) contaminated by manufactured products, particularly during the heating of plastic containers (Kubwabo et al. 2009). In the National Health and Nutrition Examination Survey (NHANES), BPA levels ranged from 0.4 to 149 μg/L (1.8–660 nM) in 92.6% of urine samples from U.S. residents ≥ 6 years of age (Calafat et al. 2008).

Exposure to BPA in humans has been implicated in the development of chronic diseases, including diabetes, asthma, and cancer (Alonso-Magdalena et al. 2010; Li et al. 2011; Midoro-Horiuti et al. 2010; Watson et al. 2010), and in causing decreased fecundity in wildlife via disrupted spermatogenesis and ovulation (Li et al. 2011; Oehlmann et al. 2009; Sohoni et al. 2001; Zhou et al. 2011). The European Food Safety Authority has set a tolerable daily intake (TDI) for BPA of 0.05 mg/kg body weight/day, a value accepted by many regulatory agencies, including the U.S. Environmental Protection Agency (1993). Because of increased concern over the safety of BPA, Health Canada (2009), and more recently the European Union (European Commission 2011) and the U.S. Food and Drug Administration (2012), have banned its use in plastic bottles for infants.

More stringent global regulations on BPA production and use have led to the development of alternative, more heat-stable bisphenol compounds (Gallar-Ayala et al. 2011; Liao et al. 2012a, 2012b). Among these alternative compounds is 4,4´-dihydroxydiphenyl sulphone, commonly known as bisphenol S (BPS). Because of the novel nature of BPS, *in vivo* toxicity studies have not been reported, nor has the ability of BPS to disrupt the actions of physiologic estrogens been explored. Several studies have tested the effects of BPS via genomic mechanisms using extremely high concentrations (concentrations unlikely to be leached from BPS-containing products). At concentrations as high as 0.1–1 mM, BPS showed only slight estrogenic activity in a 4-hr recombinant two-hybrid yeast test system (Hashimoto et al. 2001; Hashimoto and Nakamura 2000). In another such study, Chen et al. (2002) showed that 40 μM BPS had 15-fold lower genomic estrogenic activity than BPA. However, BPS was equipotent to BPA in an estrogen-response-element–driven green fluorescent protein expression system in MCF-7 breast cancer cells (Kuruto-Niwa et al. 2005). Discrepancies between these studies were attributed to species (yeast vs. mammalian) differences (Kuruto-Niwa et al. 2005). However, tissues frequently differ in responses, so this could also explain the discrepancies. No studies prior to ours have examined BPS for nongenomic mechanisms of action or at the low concentration ranges likely to be present in foods, environmental samples, or humans.

BPA can potently interfere with the actions of endogenous estrogens in pituitary cells via several types of nongenomic signaling [e.g., mitogen-activated protein kinases (MAPKs), Ca^2+^ influx] (Kochukov et al. 2009; Wozniak et al. 2005) acting via membrane estrogen receptors [mERα, mERβ, and GPER/GPR30 (G protein-coupled estrogen receptor)], and thus alter functional responses [cell proliferation, prolactin (PRL) release, and transporter function] at picomolar and subpicomolar concentrations (Alyea and Watson 2009; Jeng et al. 2010; Jeng and Watson 2011; Wozniak et al. 2005). Physiologic estrogen actions are disrupted by BPA and other XEs for both timing and magnitude of responses—enhancing or inhibiting—depending on their concentrations (Jeng et al. 2010; Jeng and Watson 2011). Introduction of a new active bisphenol compound (BPS) into the environment poses an unknown threat for signaling and functional disruptions.

In the present study we examined the effects of BPS on nongenomic signaling at concentrations that allow full assessment of potency given the nonmonotonic concentration responses we expected based on our previous studies of BPA (Jeng et al. 2010; Jeng and Watson 2011). To simulate likely exposures, we tested BPS both alone and in combination with the physiologic estrogen estradiol (E_2_). Using prototypic receptor inhibitors, we sought to identify the predominant mER through which BPS initiates nongenomic signaling. Effects of BPS on associated downstream (from MAPKs) functional end points were also examined, including changes in cell number (proliferation or decline) and caspase activations or inhibitions occurring via external stimuli (caspase 8) versus internal stimuli (caspase 9). Together, these mechanisms can contribute to alterations in cell number. Finally, we examined the effect of BPS on peptide hormone release (PRL). These measurements employed high-throughput plate immunoassays to facilitate quantitative comparisons between responses to different compounds and mixtures.

## Materials and Methods

*Cell culture*. We selected the clonal rat prolactinoma cell line GH_3_/B_6_/F_10_ on the basis of its naturally high expression of mERα (Pappas et al. 1994, 1995a), which enables it to respond robustly in tests for nongenomic signaling and functional end points. Cells were routinely subcultured with phenol red-free Dulbecco’s modification of Eagle’s medium (DMEM, high glucose; Mediatech, Herdon, VA) containing 12.5% horse serum (Gibco BRL, Grand Island, NY) and defined supplemented calf and fetal serum (Thermo Fisher, Waltham, MA) at 2.5% and 1.5%, respectively. Cells of passages 10–20 were used for these experiments.

*Concentration ranges selected*. All concentrations for time courses and dose responses were chosen based on our previous studies (Jeng et al. 2009, 2010; Jeng and Watson 2011; Kochukov et al. 2009) that demonstrated expected potencies, efficacies, and rapidity of the responses. The chosen concentrations of BPS reflect the range of concentrations likely to be found in the environment, centering on urinary levels (0.299 ng/mL or 1.2 nM), observed in Albany, New York, residents (Liao et al. 2012a, 2012b). Lower concentrations are of interest to determine how sensitive biological systems are to presumably more widespread exposure concentrations. These concentrations of other XEs were able to activate MAPKs and caspases and disrupt PRL secretion.

*Quantitative ERK (extracellular signal regulated kinase) and JNK (c-Jun-N-terminal kinase) phosphorylation assays*. To quantify phosphoactivation of ERK (pERK) and JNK (pJNK), we used a fixed cell-based immunoassay, as previously described (Bulayeva and Watson 2004). Cells (10^4^/well) were plated in 96-well plates (Corning Incorporated, Corning, NY) and allowed to attach for 24 hr. The original plating medium was then replaced with DMEM containing 1% charcoal-stripped (4×) serum for 48 hr to deprive cells of serum hormones. Medium was then removed, and cells were exposed to BPS (10^–15^ to 10^–7^ M), BPA (10^–15^ M), or E_2_ (10^–9^ M) (all from Sigma-Aldrich, St. Louis, MO) to assess time- (0–60 min) and concentration-dependent changes. Test compounds were dissolved in ethanol (EtOH), then diluted in DMEM containing 1% charcoal-stripped serum. The vehicle control was 0.001% EtOH in DMEM. To stop mER-initiated signaling, cells were fixed with a 2% paraformaldehyde/0.2% picric acid solution (Fisher Scientific, Pittsburgh, PA) at 4°C for 48 hr. Once fixed, cells were incubated with phosphate-buffered saline (PBS) containing 0.2% fish gelatin and 0.1% Triton X-100 (Sigma-Aldrich) for 1 hr at room temperature (RT) and then incubated with primary antibodies against pERK or pJNK (1:500 in PBS/0.2% fish gelatin/0.1% Triton X-100; Cell Signaling Technology, Beverly, MA) overnight at 4°C. After washing 3 times with PBS, cells were incubated with a biotin-conjugated secondary antibody (1:500 in PBS/0.2% fish gelatin; Vector Laboratories, Burlingame, CA) for 1 hr at RT. Cells were again washed 3 times in PBS, and incubated with Vectastain ABC-AP solution (50 μL/well; Vector Laboratories) for 1 hr at RT, followed by Vectastain alkaline phosphatase substrate (pNpp solution; 50 μL/well). Plates were incubated in the dark for 30 min at 37°C, and the signal for the product *para-*nitrophenol (pNp) was read at A_405_ (absorbance of 405 nm) in a model 1420 Wallac microplate reader (PerkinElmer, Boston, MA).

*Crystal violet (CV) assays*. The pNp signal was normalized to cell number, as determined by the crystal violet assay (Campbell et al. 2002). After washing 2 times with H_2_O to remove the alkaline phosphatase reaction reagents, the plates were dried at RT for 1 hr. CV solution (0.1% in H_2_O, filtered) was added (50 μL/well), incubated for 1hr at RT, and washed 4 times with H_2_O. The dye was released from the cells with acetic acid (10% in H_2_O; 50 μL/well) at RT for 30 min, and the A_590_ signal was read in the Wallac microplate reader.

*Receptor inhibitor studies*. We used prototypic selective receptor antagonists to determine the involvement of the three different types of mERs (ERα, ERβ, and GPR30) in ERK activation upon exposure to BPS (10^–14^ M). Receptor involvement in responses to BPA and NP have been determined previously (Bulayeva et al. 2005; Bulayeva and Watson 2004; Jeng and Watson 2011). Cells (10^4^/well) were plated in 96-well plates, allowed to attach for 24 hr, and treated with DMEM containing 1% 4× charcoal-stripped serum for 48 hr to deprive cells of serum hormones. Media were then removed and cells preincubated for 1 hr at 37°C with medium (50 µL) containing antagonists for ERα {MPP; 1,3-*bis*(4-hydroxyphenyl)-4-methyl-5-[4-(2-piperidinylethoxy)phenol]-1*H*-pyrazole dihydrochloride}, ERβ {PHTTP; 4-[2-phenyl-5,7-*bis*(trifluoromethyl) pyrazolo[1,5-*a*]pyrimidin-3-yl]phenol}, and GPER/GPR30 {G15; (3aS*,4R*,9bR*)-4-(6-bromo-1,3-benzodioxol-5-yl)-3a,4,5,9b-3*H*-cyclopenta[*c*]quinolone); all compounds were obtained from Tocris Bioscience (Bristol, UK) and target both membrane and intracellular versions of estrogen receptors. DMEM medium (50 μL) containing 10^–14^ M BPS was then applied to cells for 5 min, followed by fixation with a 2% paraformaldehyde/0.2% picric acid solution. The quantitative ERK phosphorylation assays were performed as described above.

*Determination of cell proliferation*. As described previously, (Jeng and Watson 2009), subconfluent cells were seeded into 96-well plates coated with poly-d-lysine (5,000 cells/well) and allowed to attach overnight. Plating medium was replaced with DMEM containing 1% 4× charcoal-stripped serum for 48 hr, and then with treatment medium containing increasing concentrations of BPS or E_2_ (10^–15^ to 10^–7^ M) alone or BPS concentrations plus 10^–9^ M E_2_. After 3 days, cells were fixed (2% paraformaldehyde/0.1% glutaraldehyde in PBS; 50 μL/well) for 20 min at RT. Cell numbers were assessed by CV assay to compare the proliferative effects of BPS at different concentrations.

*Determination of caspase activity*. Subconfluent GH_3_/B_6_/F_10_ cells were seeded into 96-well plates (5 × 10^3^/well) and allowed to attach overnight. Treatments began the next day; cells were exposed for 24 hr to DMEM medium with 1% 4× charcoal-stripped serum containing one of the following: 10^–14^ M BPS, 10^–8^ M BPS, 10^–14^ M BPS plus 10^–9^ M E_2_; 10^–8^ M BPS plus 10^–9^ M E_2_. At designated times, treatment medium was suctioned off and cells were lysed with 50 µL lysis buffer (10 mM HEPES; 2 mM EDTA; 0.1% CHAPS; pH 7.4) to which 1 mM DTT (dithiothreitol; 1:2,000, freshly prepared; Sigma-Aldrich) was added. Plates were then stored at –70°C until the assay was performed. Staurosporine (500 nM; Sigma-Aldrich) dissolved in DMSO was used as a positive control for activation of caspases 8 and 9.

For caspase assays, frozen plates were defrosted at 4°C, and assay buffer (50 mM HEPES; 100 mM NaCl; 0.1% CHAPS; 1 mM EDTA; 10% glycerol) (50 μL/well) was added. Freshly prepared 10 mM DTT and caspase 8 (Ac-IETD-AFC) or caspase 9 (Ac-LEHD-AFC) substrates (Enzo Life Sciences, Farmingdale, NY) were added to the assay buffer at final concentrations of 50 μM. Plates were then incubated in the dark at 37°C for 2 hr. The released fluorescent product, 7-amino-4-trifluoromethylcoumarin (AFC), was read using a Flexstation 3 spectrofluorometer (Molecular Devices, Sunnyvale, CA) at an excitation wavelength of 400 nm and an emission wavelength of 505 nm.

*PRL release*. Our study design and conditions were based on previous studies from our laboratory (Kochukov et al. 2009; Wozniak et al. 2005). After cells (0.5–0.7 × 10^6^) were plated into poly-d-lysine–coated 6-well plates overnight, they were hormone deprived in DMEM containing 1% 4× charcoal-stripped serum for 48 hr. Cells were then incubated for 30 min in DMEM/0.1% BSA and exposed to different concentrations of BPS alone (10^–15^ to 10^–7^ M) or in combination with E_2_ (10^–9^) for 1 min. Cells were then centrifuged at 350 × *g* for 5 min at 4°C; the supernatant was collected and stored at –20°C until radioimmunoassay (RIA) for PRL. Cells were then fixed with 1 mL of 2% paraformaldehyde/0.1% glutaraldehyde in PBS, and cell numbers were determined via the CV assay.

Concentrations of PRL secreted into the media were determined using components of the rat PRL RIA kit from the National Institute of Diabetes and Digestive and Kidney Disease and the National Hormone and Pituitary Program (Baltimore, MD). We combined 100 μL of cold standard (rat PRL-RP-3) or unknown sample with 500 μL rPRL-s-9 antiserum (final dilution of 1:437,500 in RIA buffer containing 80% PBS, 20% DMEM, and 2% normal rabbit serum) and 200 μL of ^125^I-labeled rat PRL (15,000 counts/tube diluted in RIA buffer; PerkinElmer, Wellesley, MA). The samples were then incubated and shaken overnight at 4°C. Anti-rabbit IgG was then added (200 μL of 1:9 final dilution in RIA buffer) and the samples were incubated and shaken for 2 hr at RT. After polyethylene glycol solution (1 mL; 1.2 M polyethylene glycol, 50 mM Tris, pH 8.6) was added, samples were incubated and then shaken at RT for 15 min. The samples were centrifuged at 4,000 × *g* for 10 min at 4°C, the supernatants decanted, and the pellets counted in a Wizard 1470 Gamma Counter (Perkin Elmer). PRL concentrations were calculated and normalized to CV values representing cell number.

*Statistical analysis*. Statistical analysis was performed using SigmaPlot, version 12 (Systat Software Inc., Chicago, IL). We applied one-way analysis of variance (ANOVA) to the dose- and time-dependent studies to assess the statistical significance of mean values produced by varying XE exposures. A Holm-Sidak comparison against vehicle control or against E_2_ treatment was used after the ANOVA to evaluate significance. We considered the overall α level of 0.05 to be statistically significant. In addition, we ran a Student’s *t*-test where the significance between values was borderline by one-way ANOVA, as noted in the figures.

## Results

**Figure 1:**
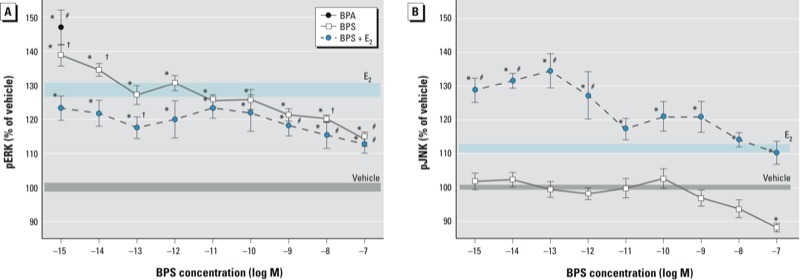
pERK (*A*) and pJNK (*B*) responses to 10^–15^ to 10^–7^ M BPS, 10^–9^ M E_2_, 10^–9^ M BPA, or 10^–9^ M E_2_ plus BPS at varied concentrations in GH_3_/B_6_/F_10_ cells. pERK (*A*) and pJNK (*B*) pNp signals measured by plate immunoassay after 5 min exposure were normalized to cell number estimates. Mean absolute absorbance values (normalized to cell number) of the vehicle control are 0.834 for ERK and 0.395 for JNK. The width of shaded areas represents means ± SEs for vehicle (gray) and E_2_ (blue); *n* = 24 over three experiments. *p < 0.05 compared with vehicle. ^#^*p *< 0.05 compared with 10^–9^ M E_2_. ^†^*p *< 0.05 compared with E_2_ using Student’s *t*-test.

Exposure of GH_3_/B_6_/F_10_ cells to BPS for 5 min caused ERK activation (Figure 1A) similar to that caused by E_2_ both here and previously (Jeng et al. 2010; Jeng and Watson 2011). The lowest tested BPS concentrations evoked a higher pERK response than did 10^–9^ M E_2_; the response steadily decreased with increasing BPS, indicating a nonmonotonic dose response (Vandenberg et al. 2012). The combination of increasing concentrations of BPS with constant 10^–9^ M E_2_ caused a lower pERK activity than did BPS alone, and was significantly lower than the nanomolar E_2_ response at the highest BPS concentrations (10–100 nM). In contrast, BPS did not produce significant pJNK activation (Figure 1B); instead the highest BPS concentration (10^–7^ M) caused deactivation significantly below vehicle levels. However, when BPS and E_2_ were administered together, JNK was strongly activated—above the level seen with E_2_ alone—and again featured a nonmonotonic dose–response curve, with the lowest concentrations evoking the largest responses.

**Figure 2:**
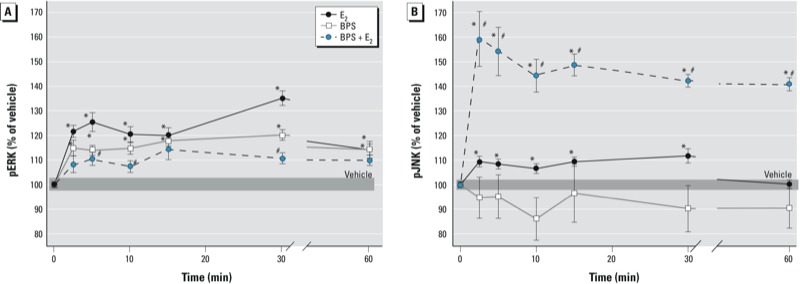
BPS (10^–14^ M) disruption of E_2_-induced (10^–9^ M) time-dependent phosphorylations of ERK (*A*) and JNK (*B*) in GH_3_/B_6_/F_10_ cells. The pNp signal for phosphorylated MAPKs normalized to the CV value for cell number is expressed as a percentage of vehicle-treated controls. Mean absolute absorbance values (normalized to cell number estimates) of the vehicle control are 0.685 for ERK and 0.395 for JNK. The width of the shaded area represents the means ± SEs of vehicle-treated cells; *n* = 24 over three experiments. *p < 0.05 compared with vehicle. ^#^*p *< 0.05 compared with 10^–9^ M E_2_.

We also examined the time dependence of these responses at optimal response concentrations (10^–14^ M BPS, 10^–9^ M E_2_; Figure 2A,B). E_2_ produced a typical oscillating two-peak ERK response, with the first peak within 5 min, followed by a second peak at 30 min (Bulayeva et al. 2004; Bulayeva and Watson 2004; Jeng et al. 2009; Jeng and Watson 2011). BPS phosphoactivated ERK within 2.5 min but did not show significant oscillation. Responses induced by BPS and E_2_ were significantly different from each other. The combination of 10^–14^ M BPS and E_2_ showed a slightly oscillating pattern, although differences between stimulated points were not significant. We have previously observed rephasing of responses due to XE combined with E_2_ (Jeng et al. 2009, 2010; Jeng and Watson 2011; Kochukov et al. 2009). Therefore, even at this very low concentration (10^–14^ M), BPS was able to disrupt the timing of the response to a physiologic estrogen. Even though 10^–14^ M BPS alone could not activate JNK at any time point tested, its combination with E_2_ dramatically enhanced the early and sustained pJNK response to E_2_ (Figure 2B).

**Figure 3:**
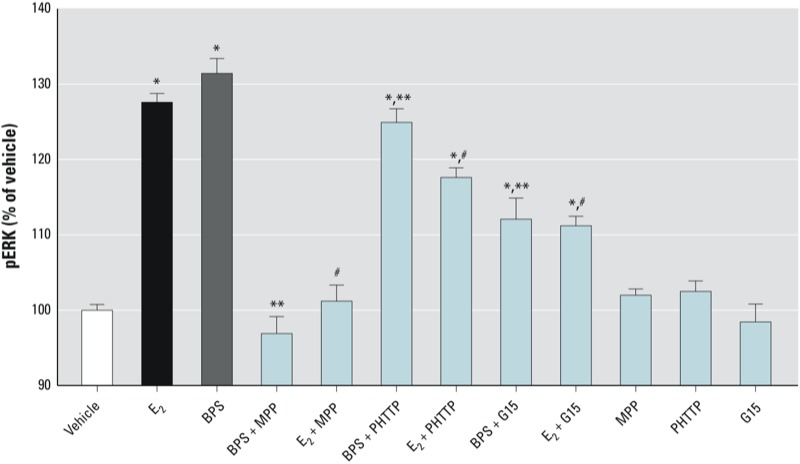
Receptor subtype–selective inhibition of BPS-induced ERK phosphoactivation in GH_3_/B_6_/F_10_ cells. Cells were pretreated for 1 hr with receptor-selective inhibitors MPP (10^–8^ M) for ERα, PHTTP (10^–7^ M) for ERβ, or G15 (10^–7^ M) for GPR30, and then treated with BPS (10^–14^ M) or the positive control, E_2_ (10^–9^ M) for 5 min, and analyzed for ERK by plate immunoassay. Values are expressed as the percentage of vehicle (mean ± SE); *n* = 16 over two experiments. For vehicle control, the mean absorbance value for pNp product, normalized to cell number estimates, was 0.743. *p < 0.05 compared with vehicle. ^#^*p* < 0.05 compared with E_2_. **p < 0.05 compared with 10^–14^ M BPS.

A prototypic chemical inhibitor for ERα (MPP, 10^–8^ M) was the most effective antagonist of E_2_ and BPS-induced responses (Figure 3). In comparison, inhibitors for ERβ (PHTTP, 10^–7^ M) and GPER/GPR30 (G15, 10^–7^ M) were much less effective in reducing the phosphoactivation of ERK by E_2_ and BPS. Therefore, mERα appears to be the predominant receptor that mediates this nongenomic response to BPS.

**Figure 4:**
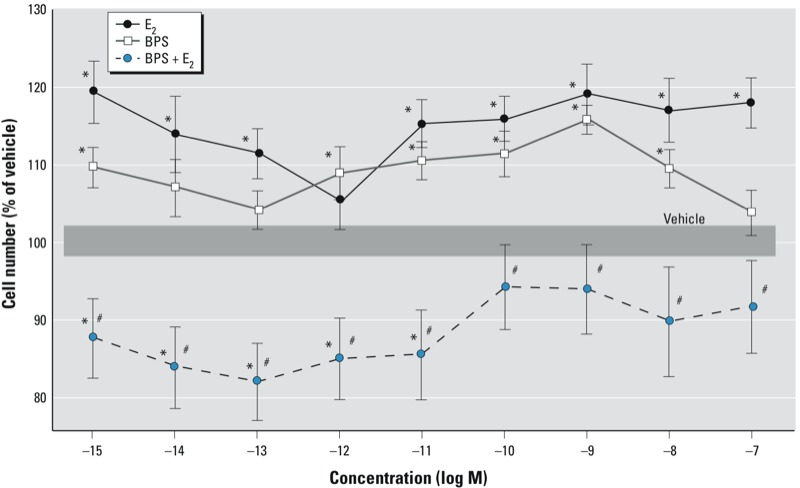
Cell proliferation in GH_3_/B_6_/F_10_ cells after 3‑day exposure to increasing concentrations of BPS or E_2_ alone, or to BPS plus a physiologically relevant concentration of E_2_ (10^–9^ M). Cell number was estimated by the CV assay (*n* = 24 over three experiments). The mean absolute absorbance value of the vehicle control is 0.299. The width of the shaded area represents the means ± SEs of vehicle-treated cells. *p < 0.05 compared with vehicle. ^#^*p* < 0.05 compared with the corresponding concentration of E_2_ alone.

After a 3-day exposure, 10^–9^ M E_2_ and BPS had similar effects on cell proliferation, causing a nonmonotonic stimulation, as we observed previously with E_2_ (Jeng and Watson 2009; Kochukov et al. 2009). The combination of BPS and E_2_ did not stimulate cell proliferation, but instead suppressed cell numbers to levels below those of cells exposed to vehicle (Figure 4).

**Figure 5:**
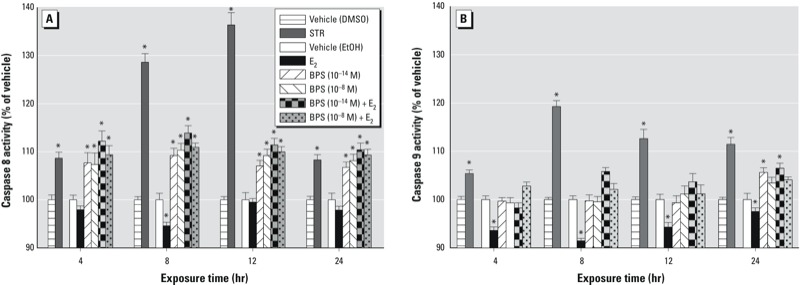
Activation of caspase 8 (*A*) and caspase 9 (*B*) in GH_3_/B_6_/F_10_ cells by BPS (10^–8^ M or 10^–14^ M), E_2_ (10^–9^ M), or BPS plus E_2_. The time dependence of activation was measured by the release of a fluorogenic substrate (AFC) expressed as a percentage of vehicle-treated controls. The absolute relative fluorescence unit values for EtOH vehicle (0.001% EtOH) are as follows: for caspase 8 at 4 hr, 63; 8 hr, 60; 12 hr, 68; and 24 hr, 70; for caspase 9 at 4 hr, 70; 8 hr, 78; 12 hr, 63; and 24 hr, 76. Staurosporine (STR; 500 nM) was used as a positive control and was compared with its own vehicle control (0.01% < 0.05 compared with vehicle.

Because decreases in cell number can be caused by apoptosis, we assayed caspases 8 and 9 to determine whether the extrinsic or intrinsic apoptotic pathways were activated. Caspase 8 was activated by both BPS and BPS plus E_2_ (10^–9^ M) at all time points tested (4–24 hr), regardless of the BPS concentration used (Figure 5A). In contrast, caspase 9 was significantly activated only at 24 hr, and by low concentrations of BPS (10^–14^ M) or its combination with E_2_ (Figure 5B). The positive control (staurosporine) was active at all times and on all caspases, as expected. Interestingly, nanomolar E_2_ alone suppressed caspase 9 activity below the level of vehicle controls at all time points, whereas inhibition below vehicle levels was observed only at the 8-hr time point for caspase 8, with some timing differences from our previous studies (Jeng and Watson 2009).

**Figure 6:**
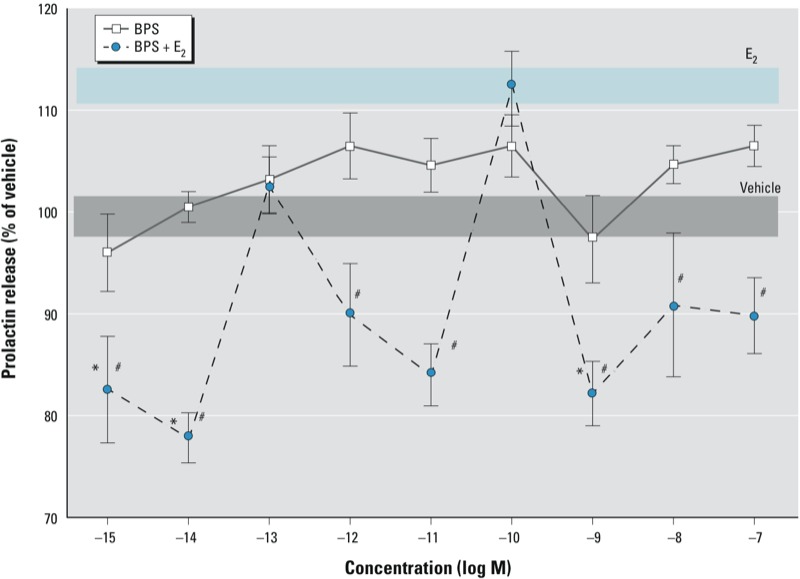
Effect of BPS on E_2_-induced PRL secretion in GH_3_/B_6_/F_10_ cells. The amount of PRL secreted for each well (counts per minute) was normalized to the CV value for cell number, and expressed as a percentage of vehicle-treated controls. The absolute value (normalized to cell number estimates) of the vehicle control is 466. The width of shaded areas represents means ± SEs for vehicle (gray) and E_2_ (blue). Values shown are means ± SEs; *n* = 24 over three experiments. *p < 0.05 compared with vehicle. ^#^*p* < 0.05 compared with 10^–9^ M E_2_.

The GH_3_/B_6_/F_10_ cell line secretes PRL in response to E_2_ and a variety of estrogenic compounds, thus making this model an excellent tool for evaluating functional responses to estrogens (Dufy et al. 1979; Jeng et al. 2009, 2010; Kochukov et al. 2009; Pappas et al. 1995b; Wozniak et al. 2005). After a typical exposure time of 1 min, BPS did not significantly increase PRL secretion but E_2_ did (Figure 6). In cells treated with BPS plus E_2_, the E_2_-induced PRL release was severely inhibited in a nonmonotonic pattern, well below that in nanomolar-E_2_-treated cells; at most concentrations of BPS, the PRL release was well below that of vehicle. The PRL release after treatment with BPS (10^–10^ M) plus E_2_ was not statistically different from the level of release caused by E_2_ alone, nor was it statistically different from vehicle because of errors for that measurement.

## Discussion

Increased scrutiny and concern by government agencies and environmental advocacy groups led to the development of potential chemical replacements for BPA, such as BPS. Although BPS is less likely to leach from plastic containers with heat and sunlight, it does still escape the polymer in small quantities under normal use (Kuruto-Niwa et al. 2005; Simoneau et al. 2011; Viñas et al. 2010). Our results show that BPS is active at femtomolar to picomolar concentrations, and can alter a variety of E_2_-induced nongenomic responses in pituitary cells, including pERK and pJNK signaling and functions (e.g., cell number, PRL release).

BPS had the same capability as E_2_ for initiating phosphoactivation of ERK across concentrations and times (Jeng et al. 2009, 2010; Jeng and Watson 2009, 2011; Kochukov et al. 2009; Wozniak et al. 2005), with lower concentrations of BPS being more effective. BPS was also equipotent to BPA in the phosphoactivation of ERK. Such nonmonotonic dose responses are controversial and have been heavily examined recently (Vandenberg et al. 2012). The fluctuation of MAPK activities with concentration and time could involve several mechanisms (Conolly and Lutz 2004; Vandenberg et al. 2012; Watson et al. 2010; Weltje et al. 2005), including receptor desensitization due to overstimulation, activation of phosphatases, and simultaneous activation of multiple signaling pathways, thereby activating proteins at different rates (Vandenberg et al. 2012; Watson et al. 2011). MAPK down-regulation is critical for preventing adverse effects of extended pathway stimulation (Hunter 1995). In our mixture studies (BPS plus E_2_), attenuation of the ERK response may protect the cell against unnecessary and perhaps dangerous estrogenic stimulation caused by the increased overall estrogenic concentration of two estrogenic compounds.

Nongenomic and functional actions initiated in this cell line were mediated predominantly by mERα. In previous studies, chemical inhibitors effective for both mERα and mERβ (ICI 187,634) also blocked ERK responses (Bulayeva et al. 2005; Bulayeva and Watson 2004). In contrast to the GH_3_/B_6_/F_10_ cells used here, GH_3_/B_6_/D_9_ pituitary cells expressing low mERα levels were unable to respond via E_2_-induced activation of MAPK signaling (Bulayeva et al. 2005; Bulayeva and Watson 2004). In the present study, our experiments with subtype-selective antagonists also demonstrated that mERα was the predominant membrane receptor mediating these responses, as we reported previously (Alyea et al. 2008; Jeng and Watson 2011), although, as in our past studies, ERβ and GPR30 also contributed to this ERK response to estrogens.

Phosphoactivation of ERK and JNK has been closely associated with opposing functional end points. For example, ERK signaling (RAF→MEK1,2→ERK1,2) is often associated with cell differentiation and growth, whereas JNK signaling is usually thought to accompany the initiation of apoptosis (Junttila et al. 2008; Meloche and Pouyssegur 2007; Nordstrom et al. 2009; Xia et al. 1995). Simultaneous phosphoactivation of ERK and inactivation of JNK by BPS, as our data show, could simultaneously stimulate proliferation and inactivate cell death, magnifying the increase in cell number (Junttila et al. 2008). Our BPS/E_2_ mixture activated both ERK and JNK, perhaps correlating with a decline we saw in cell number, if the balance of these two activities is important for the outcome. Earlier studies reported that BPS alone is capable of inducing cell proliferation in the MCF-7 cell line (Hashimoto and Nakamura 2000; Hashimoto et al. 2001; Kuruto-Niwa et al. 2005) but noted that BPS began to show cytotoxic effects at concentrations > 10^–4^ M (well above the highest concentration we tested). Therefore, the proliferative/antiproliferative responses caused by BPS can happen in multiple responsive tissues.

This is the first study to explore the ability of BPS to activate caspases. Early activation of caspase 8 (compared with caspase 9) indicates that the extrinsic pathway, which involves extracellular stimuli acting on cell-surface receptors, is the primary apoptotic pathway. The reason for later and weaker activation of caspase 9 can be explained by crossover to that pathway via a lengthy process initiated by the cleavage of Bcl2-interacting protein (BID) in the caspase 8 pathway; this results in the translocation of BID to mitochondria, where it causes later release of cytochrome *c* and subsequent activation of caspase 9 pathways (Kruidering and Evan 2000; Medema et al. 1997). We previously showed increased activation of caspase 8—but not caspase 9—in phytoestrogen-treated GH_3_/B_6_/F_10_ cells after 24 hr of treatment (Jeng and Watson 2009).

Cell survival versus cell death is determined by the balance of several cellular signaling responses, and the activation of capsases is only one of many factors. There are also discrepancies in the literature about the role of ERK and JNK activation in controlling cell number. Phosphoactivation of ERK can, for example, lead to the activation of the antiapoptotic protein Mcl-1, which binds to Bax protein and prevents its activation, thus inhibiting apoptosis (McCubrey et al. 2007). Activation of ERK has also been shown to inhibit caspase 9 upon phosphorylation (Allan and Clarke 2007, 2009; Allan et al. 2003); this is perhaps a mechanism promoting the protective effects we see with E_2_ both here and in past studies (Jeng and Watson 2009). Phosphoactivation of JNK can lead to activation of several pro-apoptotic proteins such as Bax, caspase 3, cyclin D1, Fas, and interleukin 1 (Ip and Davis 1998). However, JNK has also been linked to the activation of prosurvival pathways, with the final functional response dependent on the overall balance between ERK and JNK activities (Dhanasekaran and Reddy 2008; Sanchez-Perez et al. 1998). More examples of these conflicting outcomes need to be studied to resolve the composite contributions of MAPKs to the control of cell number.

BPA and other XEs are potent inducers of PRL release (Jeng et al. 2009, 2010; Kochukov et al. 2009; Wozniak et al. 2005); in contrast, BPS caused minimal PRL release on its own. However, BPS dramatically disrupted E_2_-induced PRL release, as do other XEs. Disturbances in the timing or amount of PRL released can lead to a variety of physiologic complications, including electrolyte imbalance, disruptions in growth and development, metabolic dysfunctions, behavioral disturbances, reproductive failure, or lactation failure. In all, PRL regulates > 300 biological functions (Bole-Feysot et al. 1998). The differences we have observed between these two structurally similar bisphenol compounds warrant future examination of structure–activity relationships for these responses.

Using urine samples collected for NHANES, Calafat et al. (2008) observed total BPA concentrations across various demographic groups in the United States, with a geometric mean of 2.6 μg/L (10 nM). In comparison, Liao et al. (2012a) determined the occurrence of BPS in humans in seven different countries, with the highest urinary geometric mean concentrations in Japan, followed by the United States (Albany, NY), with a geometric mean of 0.299 ng/mL (1.2 nM), a concentration much higher than those used in our studies. Because earlier studies focused entirely on genomic mechanisms of BPS action in which BPS was active only in the micro- to millimolar range, those effects would be relevant only to high-dose exposures such as industrial accidents.

Our study is the first to demonstrate that the BPA-substitute BPS can induce rapid nongenomic signaling in estrogen-responsive pituitary cells at low (femtomolar to picomolar) concentrations. Another cause for concern is that BPS also interferes with physiologic E_2_ signaling that leads to several functional end points. These findings highlight the need for efficient *in vitro* screening methods to pretest possible substitutes for XEs before they are deployed in manufacturing. As more related compounds are tested, we can establish a list of structural features likely associated with risks in this class of chemicals, and perhaps guide future designs away from these structures that can adversely affect human and animal health.
